# Perioperative pectoral nerve block type II and postoperative recurrence in breast cancer: a randomized controlled trial

**DOI:** 10.1186/s12893-022-01895-3

**Published:** 2022-12-30

**Authors:** Lili Yu, Xiuling Cui, Panpan Song, Chunlei Li, Haochen Zhao, Yulin Chang

**Affiliations:** Department of Anesthesiology, Cangzhou Central Hospital, No.16, Xinhua Road, Cangzhou, 061000 Hebei China

**Keywords:** Breast cancer, Pectoral nerve block type II, Postoperative recurrence

## Abstract

**Background:**

A new technique for analgesia called pectoral nerve block is widely used in surgeries of breast cancer. Pectoral nerve block type II (Pecs II) block has less influence on immunity when compared with general anesthesia method. The purpose of this research is to demonstrate whether Pecs II block has influence on the recurrence of breast cancer after surgical operation.

**Methods:**

526 breast cancer patients were recruited in this research and randomized into general anesthesia group and general anesthesia with Pecs II block group. Recurrence-free survival (RFS), distant recurrence-free survival (DRFS), and overall survival (OS) were evaluated for the two groups.

**Results:**

Based on the statistical data, only the consumption of remifentanil was dramatically reduced by the performance of Pecs II block when compared with general anesthesia method. The performance of Pecs II block had no significant influence on OS, RFS, and DRFS of breast cancer patients after surgery. ASA physical status III, TNM stage 2 + 3, and mastectomy were proved to have association with lower recurrence-free survival.

**Conclusion:**

In conclusion, the performance of Pecs II block declined the remifentanil consumption during surgery of breast cancer. Meanwhile, the performance of Pecs II block had no significant influence on the OS, RFS, and DRFS of breast cancer patients after surgical resection.

## Introduction

Breast cancer is a type of the malignant tumor with the highest incidence in women all over the world [1]. In recent decades, the resection of cancer tissues through surgical operation is the principal breast cancer therapeutic strategy. Although surgical resection is performed to eliminate most of the cancer tissues, the metastasis of breast cancer still could happen through the migration of cancer cells into blood and lymphatic system [2]. Under the immunosuppression caused by both anesthesia and surgical operation, cancer cell metastasis will lead to breast cancer recurrence [3]. Breast cancer-caused mortality is mainly the result of metastasis or recurrence and the perioperative period is thought to be decisive window [4]. Therefore, cancer recurrence after surgery is one of the biggest threats to patients with breast cancer.

Three perioperative factors participate in breast cancer recurrence after surgery. First, surgical operation inhibits cell-mediated immunity, elevates proangiogenic factor concentration, decreases antiangiogenic factor concentration, and promotes the release of malignant tissue-relative growth factors [4]. Second, the use of volatile anesthetics influences the function of immune cells and directly enhances the proliferation of cancer cell [5]. Third, opioid analgesics will suppress the function of both humoral and cellular immune, promote angiogenesis, and enhance the growth of breast cancer [6]. Currently, a new technique for analgesia called pectoral nerve (Pecs) block has been widely used in surgeries of breast cancer [7]. Through blocking the intercostal and pectoral nerves, Pecs block showed relatively less complications during surgeries [8]. Pecs II block is built up based on Pecs I block and a second injection is added in the lateral branch of intercostal nerve [9]. Pecs block is proved to enhance analgesic effect, reduce opioids consumption during surgery, and decline surgery-related chronic pain [10, 11]. Meanwhile, Pecs II block also decreases the influence on immune function when compared with general anesthesia method [12].

The selection of anesthesia method for cancer tissue resection has correlation with the recurrence of cancer. Compared with volatile anesthesia, paravertebral block does not reduce breast cancer recurrence [13]. However, the influence of Pecs II block on breast cancer recurrence after surgical operation is still unknown. In this research, we aimed to demonstrate whether Pecs II block has influence on the recurrence of breast cancer after surgical operation.

## Methods

### Patients

This randomized controlled trial was approved by the ethics committee of Cangzhou Central Hospital (2020-236-01 (Z)). This study followed the Declaration of Helsinki, Ethical Principles for Medical Research Involving Human Subjects. Written and informed consents were obtained by all the participants. Breast cancer patients prepared to receive surgery were recruited in this research. The exclusion criteria involved age < 18, history of other cancer, history of previous surgery, ASA status of IV or higher, respiratory disease, heart disease, drug allergic in surgery, having chemotherapy or radiotherapy in recent 8 weeks, usage of steroids or opioids, and alcohol dependence. After exclusion, 526 patients remained in this research. Computer-generated randomization table was employed to distribute patients into general anesthesia group (General) and general anesthesia with Pecs II block group (General + PECS-2) in a 1:1 ratio. During this research, all the participants received appropriate surgical operation and all the clinical characteristics were recorded. All breast cancer surgery was performed by the same surgeon and no prior medications were prescribed before the induction of anesthesia. This study intended to verify that whether Pecs II block has influence on the recurrence of breast cancer after surgical operation, so no interim analysis for futility was carried out.

The study was registered in Chinese Clinical Trial Registry (ChiCTR2100043039) on 04/02/2021.

### Surgery procedure

Anesthesia was induced by intravenous (i.v.) propofol (2–3 mg/kg) and sufentanil (0.1 μg/kg). Tracheal intubation was facilitated by rocuronium (0.6 mg/kg). Anesthesia was maintained through (i.v.) propofol (6–10 mg/kg). Heart rate or blood pressure was reduced by (i.v.) sufentanil (0.25–0.50 μg). Glycopyrrolate (5–10 μg/kg) and neostigmine (50 μg/kg) were employed to antagonize neuromuscular blockade.

Pecs II block was performed based on standard method before the surgical procedure. Under the lateral third of the clavicle, axillary vein and artery were identified by an ultrasound probe. Then ultrasound probe was replaced between pectoralis minor and major muscles and a needle was introduced in an oblique manner under ultrasound guidance. 10 mL 0.5% ropivacaine was injected by the needle. Then the probe was replaced to the serratus anterior muscle. Pushed the needle tip to the potential space between the pectoralis minor muscle and serratus anterior muscle then injected 20 mL 0.5% ropivacaine. The researchers for the data analysis are blind to the group allocation.

### Variables and outcomes

Clinical characteristics were recorded in this research and these variables included age, body mass index (BMI), tumor–node–metastasis (TNM) stage, American Society of Anesthesiologists (ASA) physical status, surgery type, progesterone receptor status, oestrogen receptor status, and human epidermal growth factor receptor type 2 (HER2) expression.

Outcomes included recurrence-free survival (RFS), distant recurrence-free survival (DRFS), and overall survival (OS).

### Statistical analysis

To achieve a power of 80% and a two-tailed type I error rate of α = 0.05, each unmatched group required 213 patients. Data in patient characteristics were shown as mean (Standard deviation, SD), median (interquartile ranges), or n (%). Student’s t-test and Mann–Whitney U-test were applied for the comparison. Cox proportional hazards models were used to compare hazard ratios for the two groups. OS, RFS, and DRFS were estimated using the Kaplan–Meier method the groups were compared using the log-rank test. SPSS was used for statistical analyses. P < 0.05 were considered significant.

## Results

**Fig. 1:**
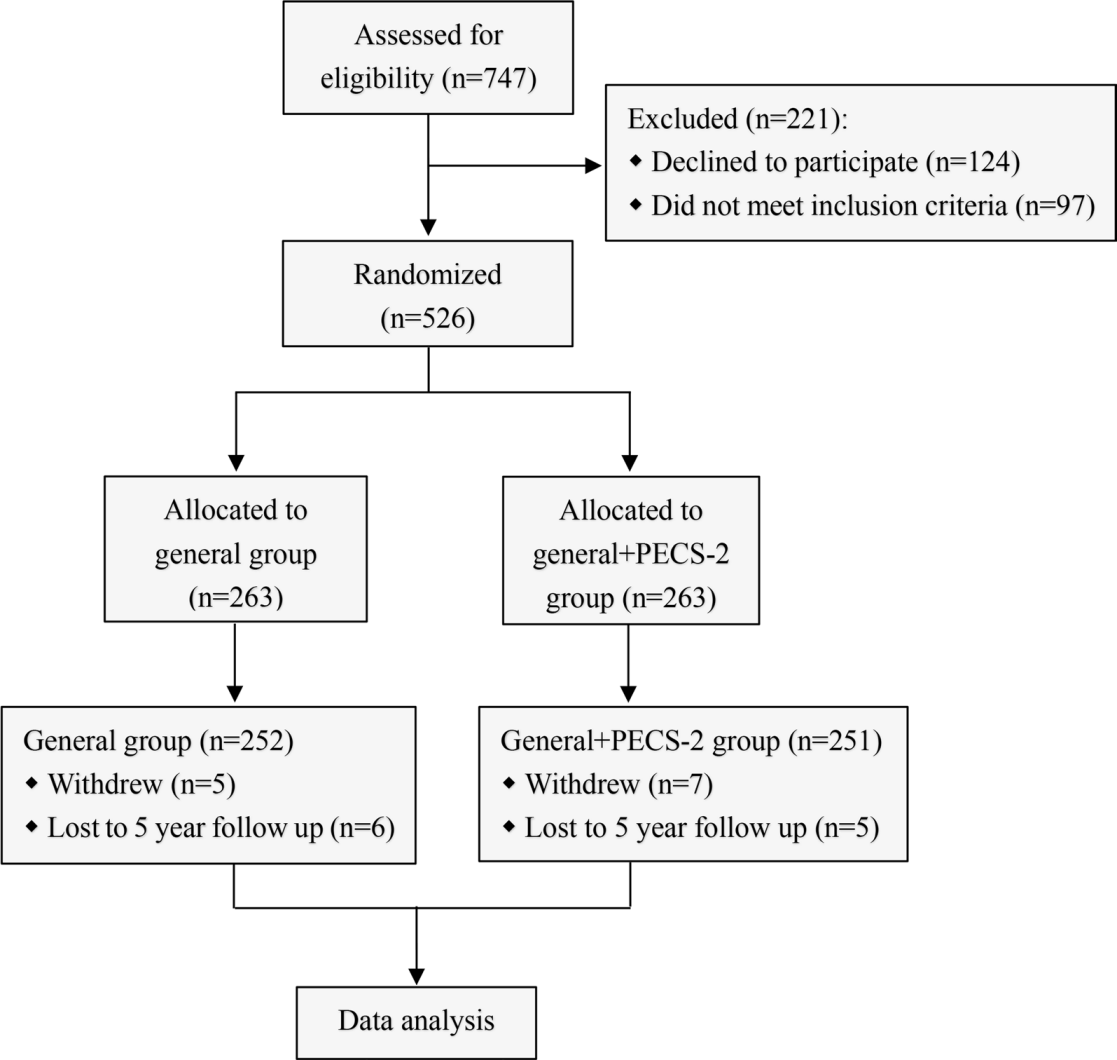
Flow diagram of the study

Flow diagram was shown in Fig. 1. 747 breast cancer patients prepared to receive surgery were recruited. There were 221 patients were excluded from this research, 124 of them declined to participate and 97 of them did not meet inclusion criteria. All the other 526 participants were randomized into general group and general + PECS-2 group. Finally, 252 participants in General group and 251 in General + PECS-2 group underwent the full study analysis.

Baseline clinical characteristics of the participants were shown in Table 1. Participants in these two groups had no differences in age, BMI, ASA physical status, TNM stage, oestrogen receptor status, progesterone receptor status, HER-2 expression, Ki67 expression, surgery type, molecular subtype, and surgical duration. Since the patients in these two groups received different anesthesia methods, intraoperative variables were also analyzed. These variables included bispectral index score, mean arterial pressure, heart rate, propofol consumption, and remifentanil consumption. Based on the statistical data, in General + PECS-2 group, only the consumption of remifentanil was dramatically reduced when compared with that in General group.

**Table 1:** Clinical characteristics of participants

	General n = 252	General + PECS-2 n = 251	P value
Age (years)	52.8 (11.3)	51.5 (10.4)	0.180
BMI (kg/m^2^)	23.6 (7.2)	24.2 (6.8)	0.337
*ASA physical status*			
I	144 (57.1)	137 (54.6)	0.436
II	75 (29.8)	87 (34.7)	
III	33 (13.1)	27 (10.8)	
Oestrogen receptor positive	210 (83.3)	199 (79.3)	0.244
Progesterone receptor positive	174 (69.0)	183 (72.9)	0.340
HER2 positive	48 (19.0)	44 (17.5)	0.660
Ki67 ≥ 30%	155 (61.5)	168 (66.9)	0.205
*Molecular subtypes*			
Luminal A	14 (5.6)	21 (8.4)	0.611
Luminal B	158 (62.7)	149 (59.4)	
HER2-enriched	33 (13.1)	31 (12.4)	
Triple negative	47 (18.7)	50 (19.9)	
*TNM stage*			
1	87 (34.5)	101 (40.2)	0.379
2	117 (46.4)	103 (41.0)	
3	48 (19.1)	47 (18.7)	
*Surgery type*			
Breast conservation	111 (44.0)	120 (47.8)	0.397
Mastectomy	141 (56.0)	131 (52.2)	
Surgical duration (min)	81 (65–124)	86 (61–136)	0.466
Intraoperative variables			
Heart rate (bpm)	74 (13)	72 (15)	0.111
MAP (mm Hg)	90 (8.9)	91 (9.2)	0.216
Bispectral index score	50.4 (11.4)	52.0 (12.0)	0.126
Propofol consumption (mg/kg/h)	7.2 (1.4)	7.0 (1.5)	0.123
Remifentanil consumption (μg/kg/h)	7.8 (3.9)	6.9 (3.3)	0.005

BMI, body mass index; ASA, American Society of Anesthesiologists; HER2, human epidermal growth factor 2; TNM, tumor–node–metastasis; MAP, mean arterial pressure

Data are reported as mean (SD), median (interquartile ranges), or n (%)

**Fig. 2:**
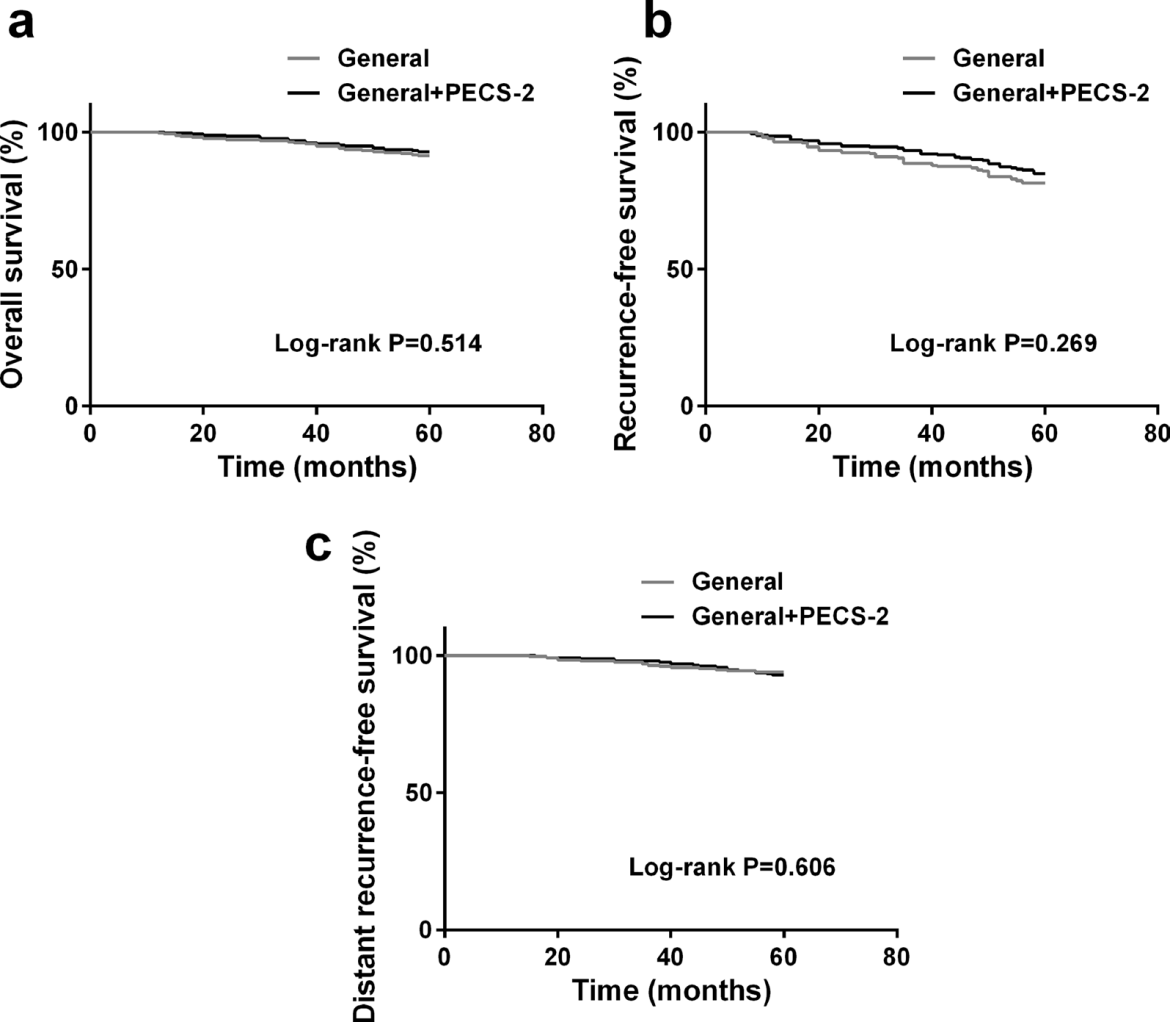
Kaplan–Meier survival analysis for **a** overall survival, **b** recurrence-free survival, and **c** distant recurrence-free survival

Through the Kaplan–Meier survival curves, recurrence-free survival (RFS), distant recurrence-free survival (DRFS), and overall survival (OS) in General group and General + PECS-2 group were analyzed (Fig. 2a–c). The performance of Pecs II block had no significant influence on OS, RFS, and DRFS of breast cancer patients after surgery.

The comparison between General group and General + PECS-2 group using Kaplan–Meier technique and multiple Cox regression analysis on the outcomes (OS, RFS, and DRFS) was shown in Table 2. Hazard ratios (HRs) in multivariate analysis were adjusted for age, ASA physical status, TNM stage, surgery type, progesterone receptor status, oestrogen receptor status, and HER2 status. The result from Cox regression showed no significant differences between these two groups for the three outcomes: OS (HR, 0.86; 95% confidence interval [CI], 0.52–1.60; P = 0.602), RFS (HR, 0.90; 95% CI, 0.76–1.32; P = 0.325), DRFS (HR, 1.29; 95% CI, 0.65–2.31; P = 0.414).

**Table 2:** Cox regression analysis for breast cancer outcomes of General + PECS-2 group vs General group

	Mortality or recurrence/total	Unadjusted		Adjusted	
		HR (95% CI)	P value	HR (95% CI)	P value
*OS*					
General	21/252 (8.3%)	1 (ref)		1 (ref)	
General + PECS-2	18/251 (7.2%)	0.81 (0.44–1.51)	0.463	0.86 (0.52–1.60)	0.602
*RFS*					
General	47/252 (18.7%)	1 (ref)		1 (ref)	
General + PECS-2	38/251 (15.1%)	0.79 (0.51–1.21)	0.193	0.90 (0.76–1.32)	0.325
*DRFS*					
General	15/252 (6.0%)	1 (ref)		1 (ref)	
General + PECS-2	18/251 (7.6%)	1.20 (0.60–2.23)	0.654	1.29 (0.65–2.31)	0.414

OS, overall survival; RFS, recurrence-free survival; DRFS, distant recurrence-free survival

HRs in multivariate analysis were adjusted for age, ASA physical status, TNM stage, surgery type, oestrogen status, progesterone status, and HER2 status

To evaluate the correlation between clinical characteristics and RFS, multivariable Cox regression analysis was constructed and results were shown in Table 3. Multivariable Cox regression demonstrated that poorer recurrence-free survival had no significant correlation with age, oestrogen receptor status (HR, 0.77; 95% CI, 0.51–1.25, P = 0.236), progesterone receptor status (HR, 0.92; 95% CI, 0.69–1.25, P = 0.526), and HER2 status (HR, 1.22; 95% CI, 0.80–1.83, P = 0.331). However, lower recurrence-free survival was proved to have association with ASA physical status III (HR, 2.14; 95% CI, 1.05–3.63, P = 0.042), TNM stage 2 + 3 (HR, 3.48; 95% CI, 1.96–5.47, P = 0.009), and mastectomy (HR, 1.77; 95% CI, 1.21–2.36, P = 0.041).

**Table 3:** Univariable and multivariable Cox regression analysis for recurrence-free survival (n = 503)

	Unadjusted		Adjusted	
	HR (95% CI)	P value	HR (95% CI)	P value
*Age (years)*				
< 40	1 (ref)		1 (ref)	
40–49	0.81 (0.51–1.16)	0.177	0.96 (0.69–1.28)	0.476
50–59	0.67 (0.49–0.93)	0.228	0.74 (0.52–1.06)	0.137
≥ 60	0.59 (0.38–0.86)	0.039	0.61 (0.41–1.02)	0.062
*ASA physical status*				
I	1 (ref)		1 (ref)	
II	1.19 (0.88–1.36)	0.571	1.23 (0.93–1.44)	0.274
III	1.89 (1.11–3.24)	0.035	2.14 (1.05–3.63)	0.042
*TNM stage*				
1	1 (ref)		1 (ref)	
2 + 3	3.72 (2.18–5.65)	< 0.001	3.48 (1.96–5.47)	0.009
*Surgery type*				
Breast conservation	1 (ref)		1 (ref)	
Mastectomy	2.11 (1.48–2.81)	0.028	1.77 (1.21–2.36)	0.041
*Oestrogen receptor*				
Negative	1 (ref)		1 (ref)	
Positive	0.62 (0.48–0.81)	0.068	0.77 (0.51–1.25)	0.236
*Progesterone receptor*				
Negative	1 (ref)		1 (ref)	
Positive	0.59 (0.46–0.77)	0.055	0.92 (0.69–1.25)	0.526
*HER2*				
Negative	1 (ref)		1 (ref)	
Positive	1.37 (0.88–2.04)	0.114	1.22 (0.80–1.83)	0.331

ASA, American Society of Anesthesiologists; TNM, tumor–node–metastasis; HER2, human epidermal growth factor 2

HRs in multivariate analysis were adjusted for age, ASA physical status, TNM stage, surgery type, oestrogen status, progesterone status, and HER2 status

## Discussion

In this randomized controlled trial, we demonstrated that Pecs II block had less remifentanil consumption during surgery when compared to general anesthesia method. Meanwhile, the performance of Pecs II block had no significant influence on the OS, RFS, and DRFS of breast cancer patients after surgical resection. Patients randomly distributed general anesthesia with Pecs II block had cancer recurrences recorded at almost the same rate as did those distributed general anesthesia. Long-term outcomes (up to 5 years after surgery) was not influenced.

The selection of anesthesia method for cancer tissue resection is crucial for the therapy since it has correlation with the recurrence of cancer [14]. Although the performance of resection is capable for removing most of the cancer tissue, some cancer cells remain in the body of patient [15]. For those cancer cells, their survival and spread could be triggered by the stress caused by surgical performance and the immunosuppression induced by anesthesia. Neuroendocrine stress is induced by surgery, inhibits the production of several different kinds of immune stimulating cytokines, and enhances anti-inflammatory cytokine level [16]. In addition to the immunosuppression effects, surgical performance also promotes cancer cell metastasis and angiogenesis [3]. The use of anesthetics was also proved to have inhibition function on cell-mediated immunity and promotion function on tumor metastasis and development [17, 18].

Sevoflurane increases the proliferation, migration and invasion of estrogen receptor (ER)-positive breast cancer cells, as well as ER-negative cells [19]. Isoflurane is associated with increased prostate cancer cell proliferation and migration and apoptotic resistance in human colon cancer cells [20, 21]. During lung cancer progression, opioids have the potential direct effect on µ-opioid receptor through growth factor-signaling proliferation, migration, and epithelial–mesenchymal transition [22]. Clinical doses of morphine promote angiogenesis and tumor progression in ER-negative breast cancer cells [23].

Patients with breast cancer scheduled for resection experience moderate to severe postoperative pain [24]. Pecs II block was employed to perform analgesia [25]. Pecs II block is guided by ultrasound and composed of an anesthetic injection below the serratus anterior and pectoralis minor muscles and an anesthetic superficial injection between the minor and major pectoralis muscles [26]. Long thoracic nerve and thoracic intercostal nerves were anesthetized by the first injection and medial and lateral pectoral nerves were anesthetized by the second injection [26]. Recently, the function of Pecs II block in reducing postoperative pain and analgesic dose during the first 24 h after breast surgery was demonstrated [27]. The selection of anesthesia method for cancer tissue resection has correlation with the recurrence of cancer. In breast cancer patients, whether the performance of Pecs II block has influence on the postoperative recurrence is still unknown.

Through randomization process, these important baseline clinical characteristics were equally distributed between General group and General + PECS-2 group. Pecs II block was performed based on standard method before the surgical procedure. In both groups, all the participants received general anesthesia during the surgery. In addition to the clinical characteristics listed in the text, genetic and other characteristics which have potential to affect cancer recurrence were not obtained in this research.

Intraoperative variables in these two groups were also recorded and analyzed. These variables consist of heart rate, MAP, bispectral index score, propofol consumption, and remifentanil consumption. It has been reported that the use of Pecs II block during anesthesia significantly reduced the consumption of remifentanil in patients with breast cancer [12]. Another study also had a similar observation that Pecs II block together with preoperative ropivacaine administration significantly declined remifentanil consumption during surgery [28]. As a consequence of the negative effects of anesthetics on the immunity of patients, less use of opioids such as remifentanil may have benefits to the outcomes of the cancer patients. However, evidences have proved that the use of opioids has no significant association with outcomes after breast cancer surgery and long-term prognosis is not significantly affected by perioperative opioid administration [12]. Another large prospective population-based cohort study also demonstrates there is no correlation with opioids administration and breast cancer recurrence [29]. In this research, we also found that patients in General + PECS-2 group had a significant lower remifentanil consumption than those in General group during surgical operation.

The recurrence of cancer after surgical operation is influenced by the complex interaction between the immune function of the patient, tumor biology, and the performance of local and systemic therapy to eradicate microscopic residual disease [30]. For breast cancer surgery, when compared to propofol-based anesthesia, sevoflurane-based anesthesia has a higher risk of cancer recurrence but showed no difference in initial 5 years’ survival rate [31]. The choose of total intravenous anesthesia or balanced anesthesia in surgery also has no influence on breast cancer recurrence [32]. The comparison between inhalation anesthesia and total intravenous anesthesia further demonstrates that anesthetic type has no association with overall survival or recurrence-free survival [33]. In this research, we investigated whether the performance of Pecs II block had influence on the recurrence of breast cancer after surgical operation. Through Kaplan–Meier survival analysis and Cox regression analysis, overall survival, recurrence-free survival, and distant recurrence-free survival did not differ by the use of Pecs II block. Thus, perioperative Pecs II block will not enhance the postoperative recurrence in breast cancer patients.

It is revealed that several different clinical characteristics are associated with high risk of recurrence after breast cancer surgery. Evidences have proved that ASA physical status, TNM stage, and type of surgical procedure have strong correlation with breast cancer recurrence [33, 34]. In this research, multivariate analyses revealed that recurrence-free survival was not associated with age, oestrogen status, progesterone status, and HER2 status. On the contrary, the performance of mastectomy, TNM stage 2 and 3, and ASA physical status III were significantly associated with breast cancer recurrence.

There were a few limitations in this study. First, genetic and other characteristics that potentially could affect cancer recurrence were not analyzed in this research. Another limitation was that the medical advances that took place during our relatively long study period could not take into account. Third, since this research was executed in a single center, multicenter studies should be performed to further investigate this conclusion. Forth, we only analyzed perioperative anesthesia method, however, further medical treatment, oncologists, and radiation therapy, which are potential confounding factors, were varied. It should mean a lot to analyze the effect of adjuvant therapies in future work.

## Conclusion

In conclusion, the performance of Pecs II block declined the remifentanil consumption during surgery of breast cancer. Meanwhile, the performance of Pecs II block had no significant influence on the OS, RFS, and DRFS of breast cancer patients after surgical resection.

## Data Availability

The datasets used and/or analysed during the current study available from the corresponding author on reasonable request.

## Abbreviations

RFS: Recurrence-free survival
DRFS: Distant recurrence-free survival
OS: Overall survival
